# Concurrent primary repair of obturator nerve transection during pelvic lymphadenectomy procedure via laparoscopical approach

**DOI:** 10.1016/j.ijscr.2018.10.081

**Published:** 2018-11-13

**Authors:** Cengiz Andan, Mehmet Sait Bakır, Serhat Şen, Şerif Aksin

**Affiliations:** a TC Ministry of Health, Health Sciences University, Gazi Yaşargil Diyarbakır Training and Research Hospital, Obstetrics and Gynecology, Diyarbakir, Turkey; b TC Ministry of Health, Health Sciences University, Gazi Yaşargil Diyarbakır Training and Research Hospital, Gynecology Oncology Clinic, Diyarbakir, Turkey

**Keywords:** Gynecologic endoscopy, Lymphadenectomy, Obturator nerve, Repair

## Abstract

**Introduction:**

Obturator nerve is barely injured during gynecological surgeries. The risk for obturator nerve injury is increased during pelvic lymphadenectomy procedures of gynecological malignancies. In case of any obturator nerve injury, surgical management involve laparoscopic approaches suchas end-to-end anastomosis in very early period.

**Case:**

A 63-year-old woman, with G3P3, presented with the complaint of abnormal uterine bleeding. She was diagnosed with stage IA endometrial adenocarcinoma. obturator nerve was transected during obturator lymph node dissection in the right side during retroperitoneal lymph node dissection. Subsequently end-to-end anastomosis of the thermally injured areas was performed by epineural sutures. Any significant loss of functions in adductor muscle wasn't observed in the postoperative period. Likewise, any permanent neurological finding wasn't developed at the end of postoperative 6th month.

**Discussion:**

During pelvic lymphadenectomy; obturatory nerve, especially the proximal part, should be concerned. Early intraoperative repair should be performed in case of possible nerve injuries.

**Conclusion:**

Immediate laparoscopic repair is possible in full-thickness injury of obturator nerve, occurred in a gynecological surgery and results with rapid and complete neurological recovery.

## Highlights

• Laparoscopic early stage nerve repair is a feasible method in gynecological surgery.
• Early nerve repair, short-term clinical and long-term neurological consequences are usually curative.
• In laparoscopic gynecologic malignancy surgery, laparoscopic management of complications is a minimally invasive procedure.

## 1 Introduction

Obturator nerve is barely injured during gynecological surgeries. The risk for obturator nerve injury is increased during pelvic lymphadenectomy procedures of gynecological malignancies In case of any obturator nerve injury, surgical management options involve laparoscopic and transabdominal approaches [1]. Here, we presented an obturator nerve injury of full-thickness transection with thermal damage caused by 5-mm laparoscopic Maryland-Style grasper dissector (Ligasure), occurred during pelvic lymph node dissection in a patient whom underwent laparoscopic hysterectomy and pelvic lymphadenectomy for endometrial cancer. The transected nerve was repaired immediately via laparoscopic approach during the same session. The aim of current study is to demonstrate importance of very early nerve repair in case of a neural injury happened during laparoscopic pelvic lymphadenectomy in gynecological cancers. In this study; this golden period of first hours, after injury, is associated with complete nerve recovery [2]. This study was reported to be consistent with a Case Report of Surgery, Guidelines (SCARE) criteria [3].

## 2 Case report

**Fig. 1:**
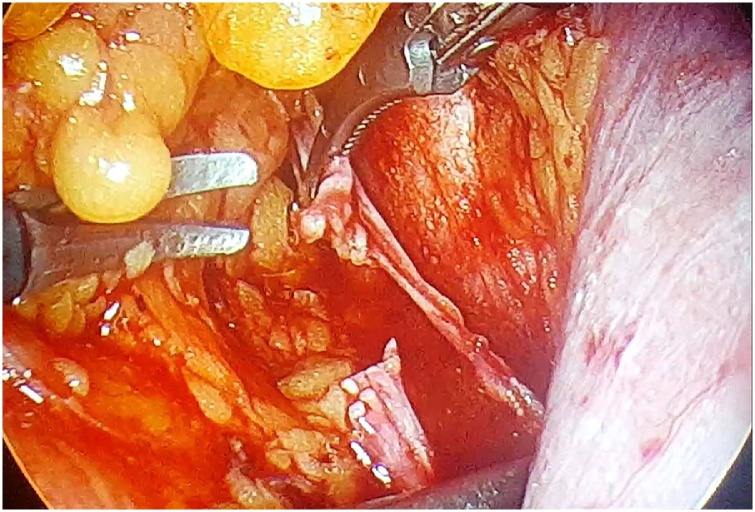
Complete transection of obturator nerve.

**Fig. 2:**
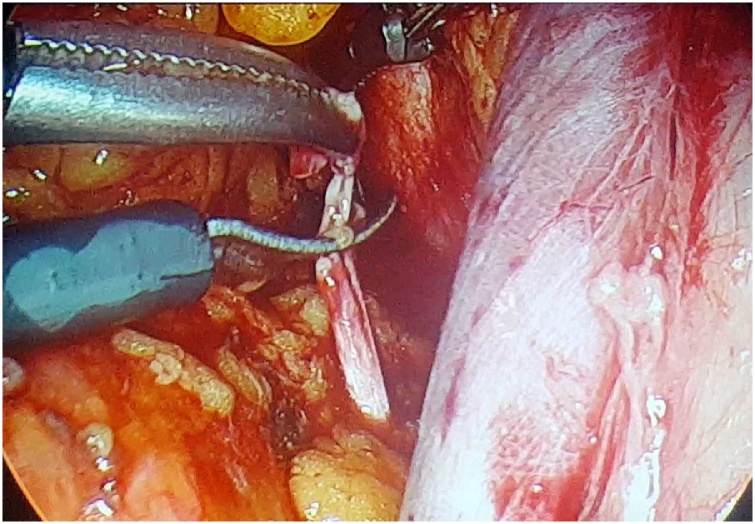
Debridement of nerve ends.

**Fig. 3:**
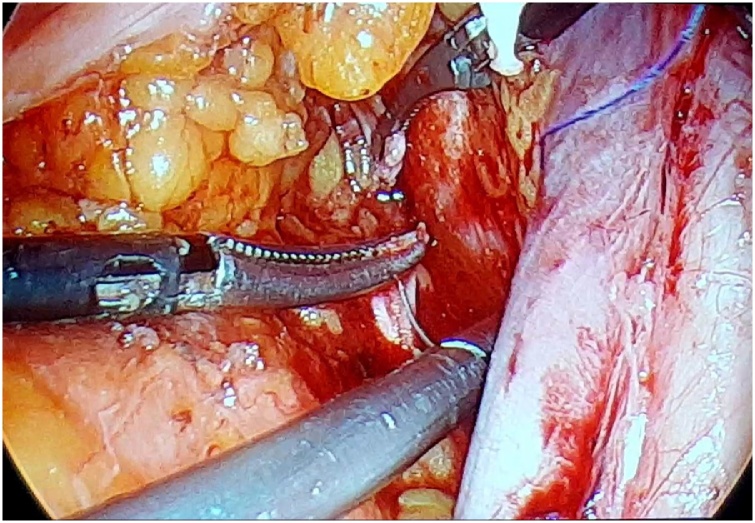
Epineural anastomosis of nerve ends.

**Fig. 4:**
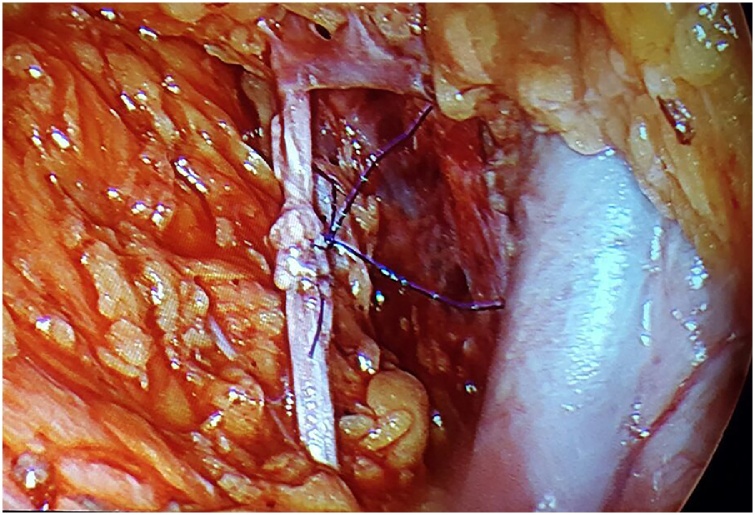
Intraoperative epineural end-to-end anastomosis.

A 63-year-old, G3Y3, postmenopausal, morbid obese woman presented to the outpatient clinics with the complain of vaginal bleeding. Physical examination was compatible with atrophy in vulva, vagina and cervix. Pap smear test was reported as negative. Transvaginal sonography revealed endometrial thickness of 12 mm. Following endometrial biopsy was reported as atypical, complex endometrial hyperplasia. Thus, laparoscopic hysterectomy and bilateral salpingo-oophorectomy and omental biopsy were performed. 10 mm telescope and advanced bipolar energy modalities were used during surgery. First, 10 mm trocar was directly inserted through subumbilical incision of 1 cm. laparoscope was inserted following insufflation of 3–4 l co2. Three 5-mm-trocars were inserted abdominally through 2 ipsilateral inguinal and single contralateral inguinal incisions. Rumi (R) II uterine manuplator was used for uterine manipulation. The frozen sections of hysterectomy specimen was compatible with a myometrial invasion more than 1/2. Therefore, bilateral pelvic lymphadenectomy was processed. We recognized that the right obturator nerve was transected accidentally by 5-mm Ligasure(R) during right obturator lymph node dissection (Fig. 1). It was seen that the nerve was transected in a full-thickness manner, besides thermal injury was occured at nerve ends. Following debridement (1 mm in size) of thermally injured areas,the nerve ends were reapproached (Fig. 2). An end-to-end reanastomosis without tension was performed by epineural sutures (4-0 polypropylene) via laparoscopy (Fig. 3, Fig. 4). The duration of repair was 21 min, while overall operation session was 180 min. Volume of blood loss was 150 mL. No marked loss of adductor function was observed during early postoperative period. The patient was discharged on day 3, postoperatively. On month 2 of the operation, the patient reported numbness at medial aspect of thigh and minimal difficulty in climbing upstairs. Complete clinical recovery was detected on month 6. No neurological deficit was detected in the muscle group innervated by obturator nerve in her EMG at the end of the 6th month, postoperatively.

## 3 Discussion

Obturator nerve arises from anterior portion of ventral rami of second, third and fourth lumbar nerves at lumbar plexus. It is the biggest branch arising from third lumbar nerve and distributed to skin overlying adductor muscles and thigh. It runs inferiorly through major muscular branch, crosses over obturator artery and enters thigh through superior aspect of obturator foramen along medial wall of pelvis. Then, obturator nerve is divided into anterior and posterior branches. Anterior branch accounts for sensorial innervation of hip joint and mid-thigh as well as motor innervation of superficial adductor muscles [4]. Clinically, obturator nerve injury manifests with sensorial loss at the medial aspect of thigh, pain at medial portion of groin and postoperative and ipsilateral adductor weakness [5]. Obturator nerve injury can be rarely seen following pelvic lymphadenectomy procedures in gynecological malignancies. Obturator nerve injury can occur as crush or stretching, electrocoagulation, ligation or transection of nerve during laparoscopic surgery [6]. The most important factor is the early diagnosis and repair. In our patient, nerve injury was recognized immediately and epineural repair was performed concurrently at the same session- in other words at very early period of damage. Clinically, no marked neurological deficit was observed in the early postoperative period. The patient was discharged on day 3, postoperatively. On month 2, the patient complained minimal difficulty in climbing stairs as well as numbness at the medial aspect of thigh. On month 6, no neurological deficit was reported in the muscle group innervated by obturator nerve on her EMG, meaning the complete clinical recovery of dissected nerve.

When literature was assessed, it was seen that Menderes et al. [7], Zhao et al. [8] and Göçmen et al. [9] reported nerve repair by laparoscopic epineural end-to-end anastomosis after recognition of intraoperative nerve transection. All authors reported complete recovery at the end of month 6. The learning curve is essential in laparoscopic surgery to gain laparoscopic skills in management of intraoperative complications. Given that, learning curve in laparoscopy is associated with appropriate management of complications; a meticulous nerve mobilization should be performed patiently during pelvic lymphadenectomy. The approach in pelvic lymphadenectomy should be from inferior to superior, while preserving obturator nerve in obturator fossa, that exposed with distraction of peritoneum towards medial.

besides, surgeons should be aware of possibility of such injuries intraoperatively. So that they could repair without any time loss, for the patients sake. A special attention should be directed to the proximal part of adjacent obturator nerve during lymphadenectomy procedure. In the literature, 80% of reported injuries are at proximal part whereas 20% are at distal part of the obturator nerve [10].

İn addition to competent knowledge of pelvic anatomy and elaborateness in safe dissection, another important issue to prevent obturator nerve is to expose nerves and vessels gently during dissection. Obturator nerve should be well-identified and demarcated from surrounding tissues, as it is covered by fat tissue and lymph nodes. To avoid such complications, proposed to place clips to obturator nerve in order to make the nerve visible.

## 4 Conclusion

It is important to prevent nerve injury, in addition to recognize neurological complications in early period, in gynecological laparoscopy procedures. Another important factor in appropriate management of such rare complications is the learning curve of the surgent in laparoscopy. Awareness of surgeon about anatomy of the nerves and mechanism of injury is crucial to prevent such injuries. Obturator nerve may be transected completely or partially due to thermal injury during laparoscopic pelvic lymphadenectomy. Early nerve repair, as soon as recognition of the transection, will facilitate neurological healing process and is associated with excellent results as presented in this case.

## Conflicts of interest

None of the authors has any potential conflicts of interest neither have they received any funding for this study.

## Funding

None.

## Ethical approval

Ethical approval has been exempted by Diyarbakir Womens and Child Diseases Hospitals organization.

## Consent

Written informed consent was obtained from the patient for publication of this case report and accompanying images. A copy of the written consent is attached and available for review by the Editor-in-Chief of this journal on request.

## Author contribution

**Table:** 

	CA	MSB	SŞ	ŞA	
Conception & Design of Study	X				
Data Collection			X		
Data Analysis & Interpretation		X			
Responsible Surgeon or Imager				X	
Statistical Analysis		X			
Manuscript Preparation	X			X	
Patient Recruitment	X	X	X	X	

## Registration of research studies

There was no need for a in this study.

## Guarantor

Dr. Şerif Aksin.

Dr. Cengiz Andan.

## Provenance and peer review

Not commissioned, externally peer reviewed.
